# Supplementary material for: A platform for multimodal in vivo pooled genetic screens reveals regulators of liver function
Source: bioRxiv. 2025 Feb 17:2024.11.18.624217. Preprint. [Version 3] doi: 10.1101/2024.11.18.624217 (PMC11601512; doi:10.1101/2024.11.18.624217)
Supplement: Supplement 10 [file NIHPP2024.11.18.624217v3-supplement-10.pdf]

bioRxiv preprint doi: https://doi.org/10.1101/2024.11.18.624217; this version posted February 17, 2025. The copyright holder for this preprint (which was not certified by peer review) is the author/funder, who has granted bioRxiv a license to display the preprint in perpetuity. It is made available under aCC-BY 4.0 International license.

Supplementary Figure Legends

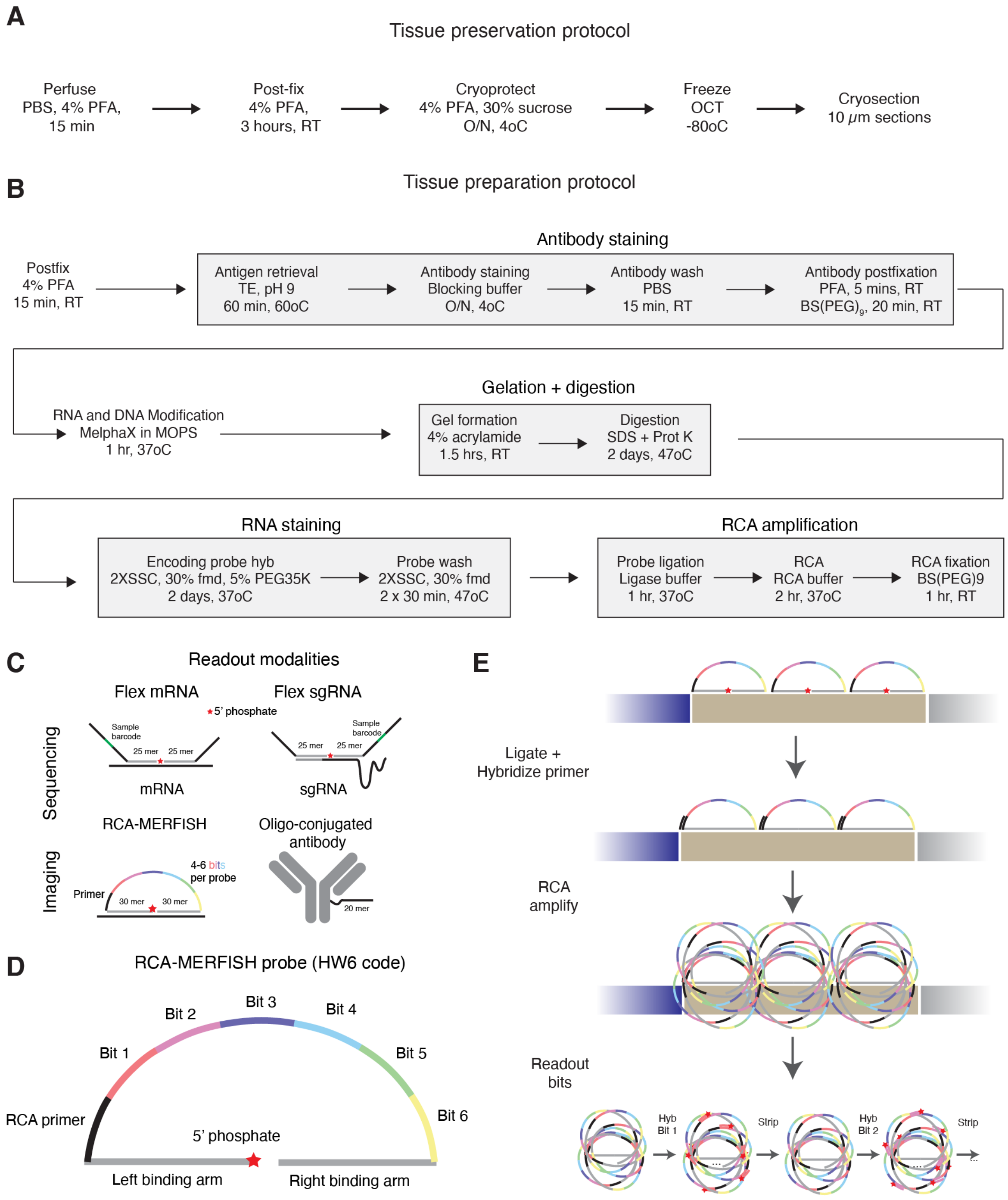

Figure S1: Workflow and optimizations for imaging-based screening

A. Experimental procedure for tissue preservation by PFA fixation and cryoprotection.

bioRxiv preprint doi: https://doi.org/10.1101/2024.11.18.624217; this version posted February 17, 2025. The copyright holder for this preprint (which was not certified by peer review) is the author/funder, who has granted bioRxiv a license to display the preprint in perpetuity. It is made available under aCC-BY 4.0 International license.

B. Detailed experimental protocol for multimodal oligo-conjugated antibody staining and RCA-MERFISH in fixed tissue.
C. Different readout modalities by imaging or sequencing
D. Diagram of padlock probe design for RCA-MERFISH with a Hamming Weight (HW) 6 code. The readout sequences (marked as bit 1 to bit 6), the presence of which determined the MERFISH code, are directly encoded in the padlock probe.
E. Diagram of RCA-MERFISH signal amplification process. Probes that have both arms hybridized to target RNA and adjacent to each other are ligated, and then the ligated probes are amplified through rolling circle amplification (RCA). After RCA, the individual bits in each amplicon are read out through fluorescent microscopy, over multiple rounds of staining with fluorescent readout probes, and then dehybridizing (‘stripping’) the probes off with a high formamide concentration wash.

bioRxiv preprint doi: https://doi.org/10.1101/2024.11.18.624217; this version posted February 17, 2025. The copyright holder for this preprint (which was not certified by peer review) is the author/funder, who has granted bioRxiv a license to display the preprint in perpetuity. It is made available under aCC-BY 4.0 International license.

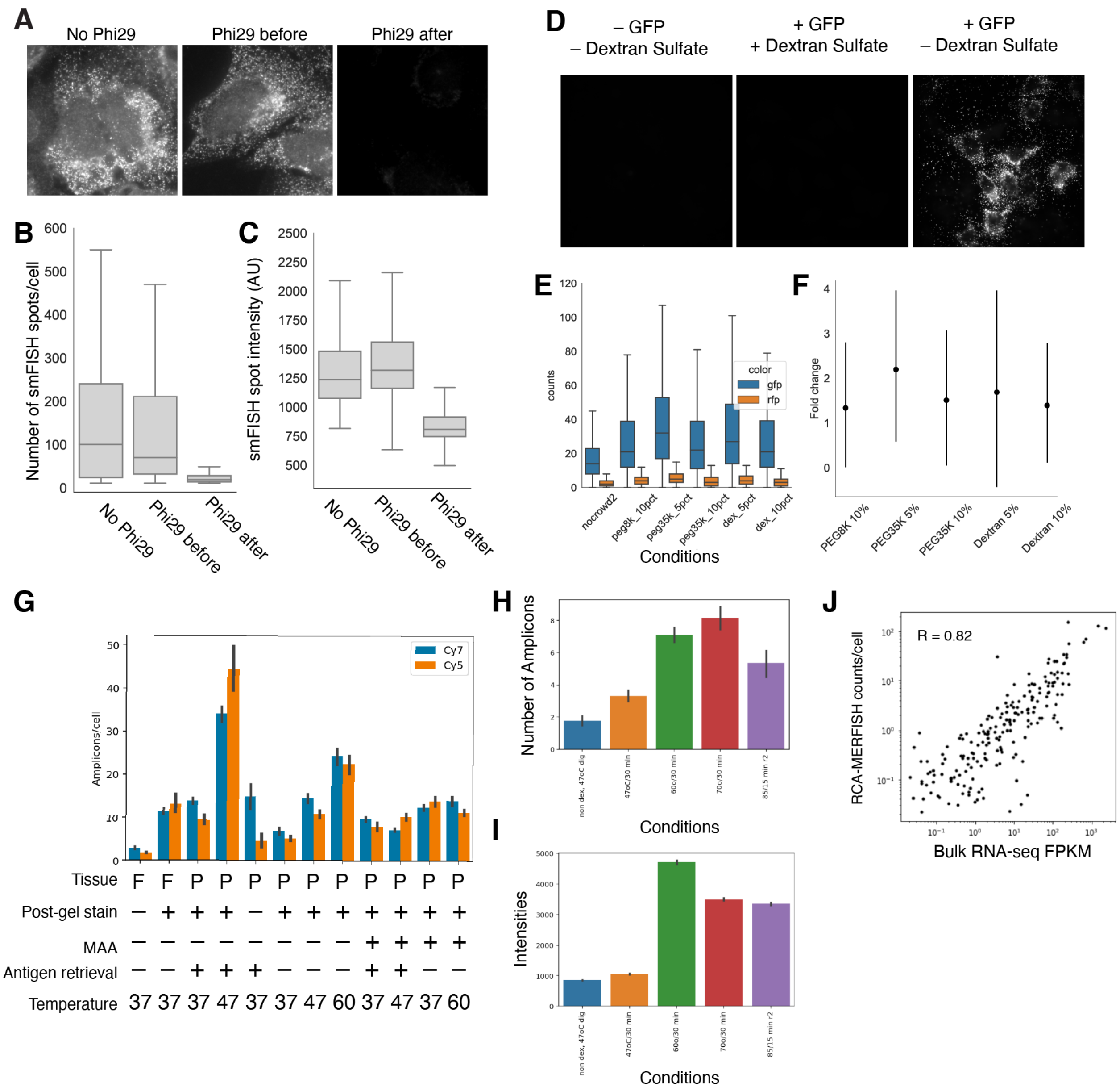

Figure S2: Optimization of RCA-MERFISH protocol

A. Phi29 used in RCA degrades ssDNA smFISH probes. Left: smFISH signal in U-2 OS cells without Phi29 treatment at 37oC for 1 hr. Center: smFISH signal in U-2 OS cells pre-treated with Phi29 at 37oC for 1 hr before FISH staining. Right: smFISH signal in U-2 OS cells treated with Phi29 at 37oC for 1 hr after FISH staining.
B. Quantification of effect of Phi29 on ssDNA smFISH probes from (A), in spots/cell.
C. Quantification of effect of Phi29 on ssDNA smFISH probes from (A), in intensity/spot.
D. Dextran sulfate inclusion in hybridization buffer inhibits Phi29 enzymatic activity. Left: amplified RCA-MERFISH padlock probe against GFP in U-2 OS cells not expressing GFP, detected by readout probes complementary to readout sequences on the padlock. Center: amplified RCA-MERFISH probe against GFP in U-2 OS cells expressing GFP, with dextran sulfate in the hybridization buffer, detected by readout probes complementary to readout

bioRxiv preprint doi: https://doi.org/10.1101/2024.11.18.624217; this version posted February 17, 2025. The copyright holder for this preprint (which was not certified by peer review) is the author/funder, who has granted bioRxiv a license to display the preprint in perpetuity. It is made available under aCC-BY 4.0 International license.

sequences on the padlock. Right: amplified RCA-MERFISH probe again GFP in U-2 OS cells expressing GFP, without dextran sulfate in the hybridization buffer, detected by readout probes complementary to readout sequences on the padlock.

E. Optimization of alternative crowding agents to dextran sulfate. Multiple additives to hybridization mixture, staining U-2 OS cells expressing either GFP or mCherry with a single probe against GFP, in terms of number of spots per cell, distinguishing GFP+ (signal) and mCherry+ (background) cells. Peg8k = Poly(ethylene glycol) average mol wt 8,000, Peg35k = Poly(ethylene glycol) average mol wt 35,000, Dex = unsulfonated dextran; all are added to the hybridization solution so the final w/v is at the indicated percent.

F. Quantification of increase in efficiency of different additives to hybridization mix from (E), relative to control (no additive to hybridization mix).

G. Optimization of RCA-MERFISH. Fresh-frozen (F) and PFA-fixed (P) tissue was tested with staining RNA after gel embedding (+ Post-gel stain) or before gel embedding (– Post-gel stain), with or without the addition of methacrylic acid NHS ester (MAA) along with MelphaX, with or without antigen retrieval, and varying the digestion temperature from 37oC to 60oC. Counts are number of amplicons per cell across the first two bits, detected in the Cy7 and Cy5 color channel, from a 120 gene RCA-MERFISH library.

H. Optimization of RCA-MERFISH across different decrosslinking conditions, quantified by amplicon counts per cell for a single bit, using an RCA-MERFISH library at low concentration (~0.1 nM/probe), hence the lower counts per cell than (G) which used ~10X higher probe concentration. The conditions tested are (1) no decrosslinking, (2) decrosslinking at 47˚ for 30 minutes, (3) decrosslinking at 60˚ for 30 minutes, (4) decrosslinking at 70˚ for 30 minutes, and (5) decrosslinking at 85˚ for 15 minutes. All decrosslinking was conducted in TE pH 9.

I. Optimization of immunofluorescence across different decrosslinking conditions, measuring total intensity per field of view for a Tomm20 antibody. The decrosslinking conditions were the same as in Figure S2H.

J. Correlation of 209 gene RCA-MERFISH (after optimization) with bulk RNA-seq from the liver.

bioRxiv preprint doi: https://doi.org/10.1101/2024.11.18.624217; this version posted February 17, 2025. The copyright holder for this preprint (which was not certified by peer review) is the author/funder, who has granted bioRxiv a license to display the preprint in perpetuity. It is made available under aCC-BY 4.0 International license.

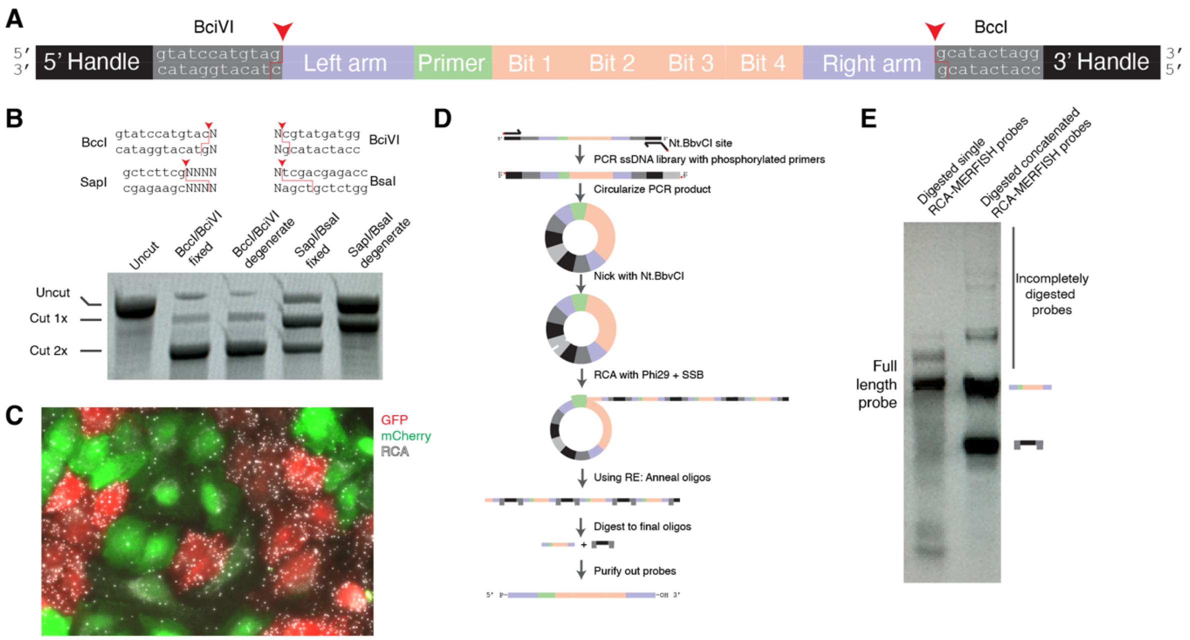

Figure S3: RCA-MERFISH padlock probe production

A. RCA-MERFISH padlock probe design for pooled oligonucleotide synthesis, prior to amplification and probe library synthesis.
B. Testing different Type IIS restriction enzymes on single restriction digested, PAGE-purified probe against GFP made through phophoramidite synthesis.
C. RCA amplicons of digested RCA-MERFISH probe against GFP in U-2 OS cells expressing either GFP or mCherry. There are many more GFP amplicons in GFP-expressing cells than there are in mCherry-expressing cells, indicating the specificity of RCA-MERFISH.
D. Probe library preparation protocol using RCA followed by restriction digestion. Femtomolar pools of oligos synthesized in arrays are amplified by PCR with phosphorylated primers containing an Nt.BbvCI site. The PCR amplicons are then circularized and nicked with Nt.BbvCI. The nick site is used to initiate rolling circle amplification, and the RCA product is then digested with BccI and BciVI after annealing on primers.
E. Comparison of digested single probes and concatenated probes produced through RCA synthesis, on a 4% agarose gel electrophoresis.

bioRxiv preprint doi: https://doi.org/10.1101/2024.11.18.624217; this version posted February 17, 2025. The copyright holder for this preprint (which was not certified by peer review) is the author/funder, who has granted bioRxiv a license to display the preprint in perpetuity. It is made available under aCC-BY 4.0 International license.

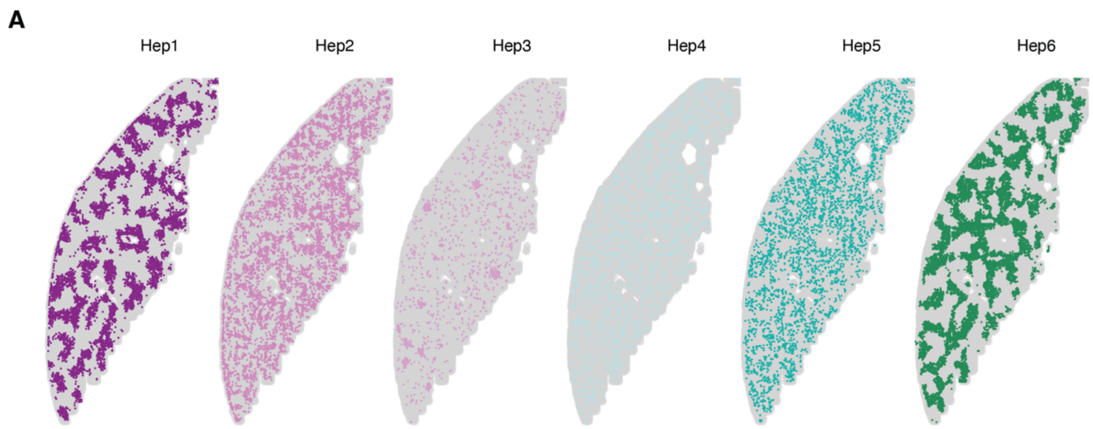

Figure S4: Hepatocyte transcriptional subtypes

A. Spatial organization of different transcriptionally-defined hepatocyte subtypes, Hep1-6.

bioRxiv preprint doi: https://doi.org/10.1101/2024.11.18.624217; this version posted February 17, 2025. The copyright holder for this preprint (which was not certified by peer review) is the author/funder, who has granted bioRxiv a license to display the preprint in perpetuity. It is made available under aCC-BY 4.0 International license.

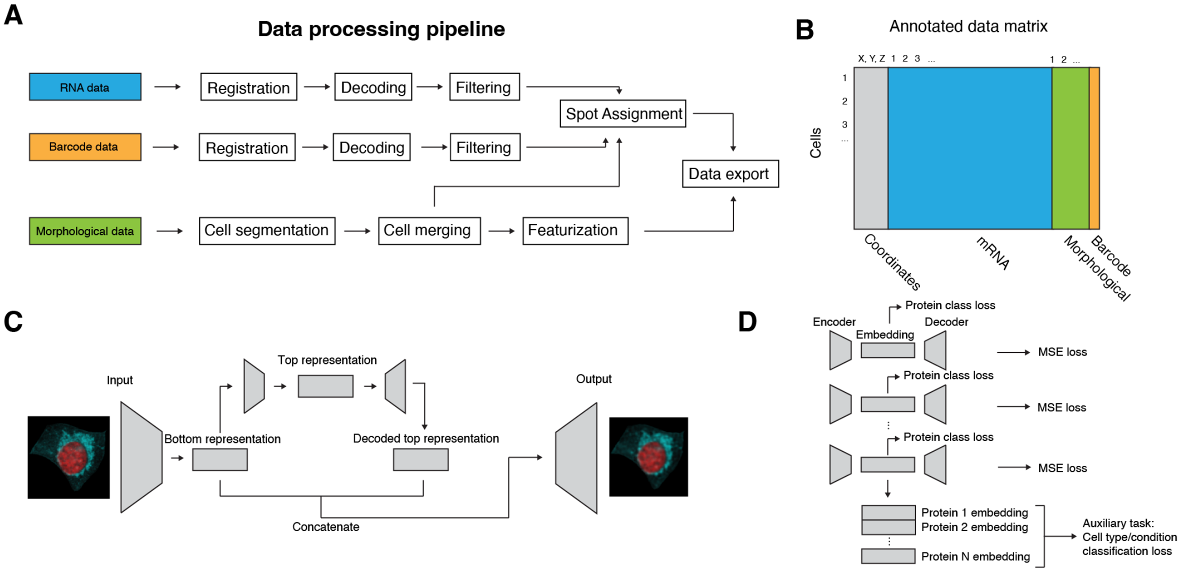

Figure S5: Data processing pipeline and deep learning model architecture

A. Diagram of data processing pipeline. Each panel of barcodes, endogenous RNA or morphological data (protein or RNA) that is measured is collected back-to-back in the same experiment and then processed in parallel. The RCA-MERFISH data is processed by first registering to common fiducials across multiple rounds, then decoding the identity of individual molecules. The molecules are then filtered using machine learning on features of molecules (mean intensity, size, variance, difference between mean on- and off-bit intensity), to obtain a final 5% false positive rate that decode to a blank barcode. In parallel, the polyA and Na+/K+ ATPase channels of the morphological data are used to segment cells, which are then merged to eliminate duplicates of the same cells segmented in multiple fields of view. The cell segmentations are used to assign molecules to individual cells for quantification, and then the morphological channels are used with the segmentation mask to export the final images and per-gene quantification of expression for each cell.
B. Diagram of final annotated data matrix combining all features.
C. High-level diagram of VQ-VAE network across all channels. An input image is put into an artificial neural network that attempts to reconstruct the same image after passing the image through a low-dimensional bottleneck. In this case, two separate representations are created (top- and bottom-level) that attempt to capture different scales of features in the image. The bottom representation is formed first through one network, then further compressed to form a top representation with a separate autoencoder. The two representations are then concatenated and passed through a final network to reconstruct the original image.
D. Detail of VQ-VAE network for each individual channel, trained simultaneously. A separate embedding is created for each morphological channel at the same time, using a mean squared error (MSE) loss to determine the accuracy of reconstruction. For each morphological channel, the embedding is used in a classification task to predict the identity of the protein or RNA being represented. The embeddings for each morphological channel are then concatenated and used to predict higher-level information about the type or state of each cell. When added to the overall

bioRxiv preprint doi: https://doi.org/10.1101/2024.11.18.624217; this version posted February 17, 2025. The copyright holder for this preprint (which was not certified by peer review) is the author/funder, who has granted bioRxiv a license to display the preprint in perpetuity. It is made available under aCC-BY 4.0 International license.

training loss, these auxiliary predictive tasks are intended to constrain the representations that are formed by the VQ-VAE networks to capture salient features that discriminate different morphological channels and cell types or states.

bioRxiv preprint doi: https://doi.org/10.1101/2024.11.18.624217; this version posted February 17, 2025. The copyright holder for this preprint (which was not certified by peer review) is the author/funder, who has granted bioRxiv a license to display the preprint in perpetuity. It is made available under aCC-BY 4.0 International license.

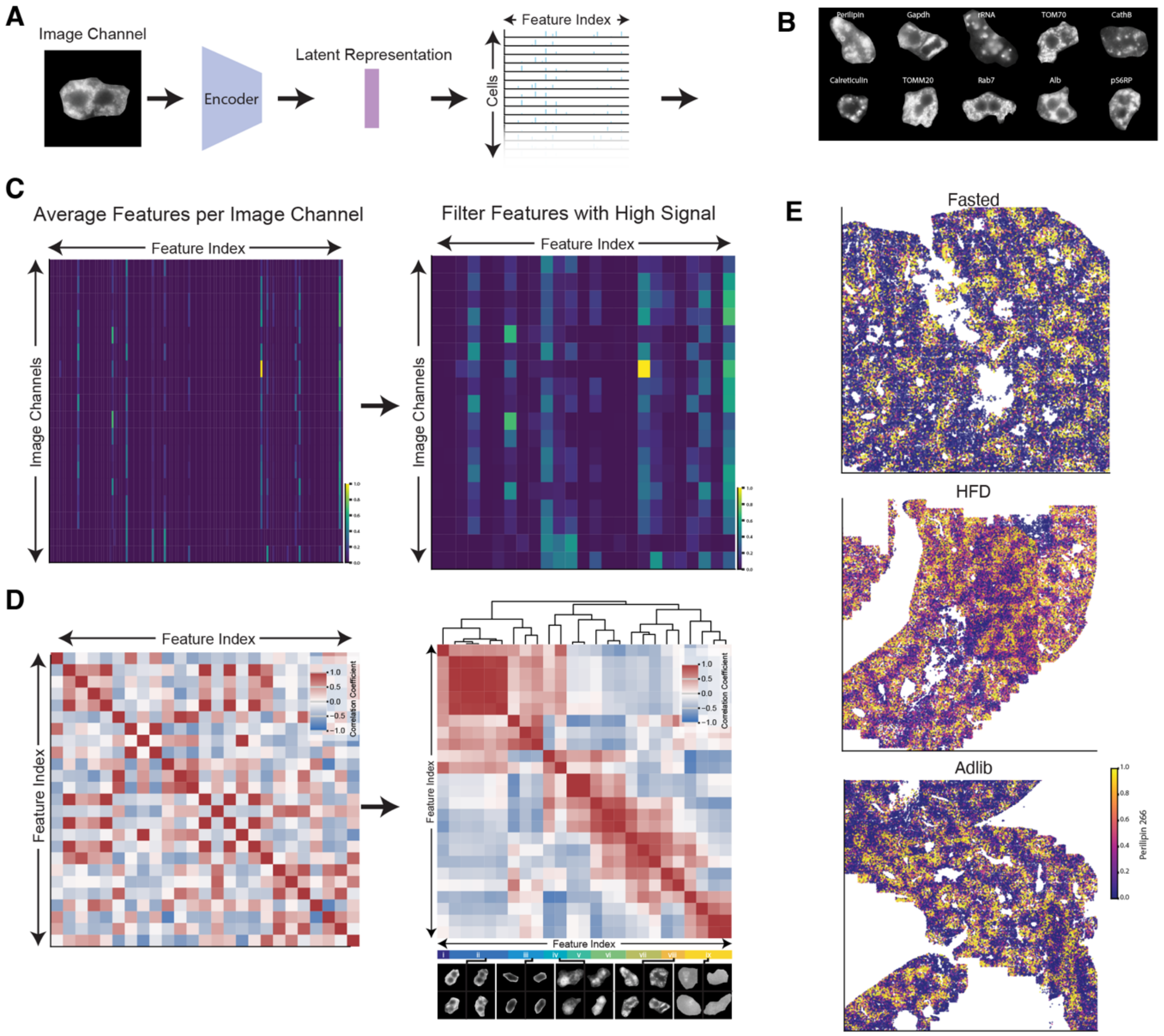

Figure S6: Analysis of image features from deep learning embedding

A. Diagram of transformation of individual imaging channels into cell by feature representations. Each image channel for each cell is reduced to 512-dimensional vector. Here, we consider each dimension a feature.
B. Examples of protein channel images that have high values for feature 266 (locally concentrated expression) from Class viii (as described below in (D)). This shows that the same protein/RNA feature measures the similar spatial patterns across different protein/RNA channels and cells.
C. Heatmap of average feature weights across different imaging channels, with all features shown (left) or only features with high weight scores (high signals) shown (right).
D. (Left) Heatmap of the pairwise correlation between high-signal features across cells. (Right) This heatmap is reordered through hierarchical clustering to reveal features that correlate strongly. Nine classes of features are manually identified and visualized. Each class of features captures similar spatial patterns. Cells with high weight scores of features from each class are displayed, including example classes (ii) cells with two Nuclei, (iii) signal enriched at cell membrane, (iv)

bioRxiv preprint doi: https://doi.org/10.1101/2024.11.18.624217; this version posted February 17, 2025. The copyright holder for this preprint (which was not certified by peer review) is the author/funder, who has granted bioRxiv a license to display the preprint in perpetuity. It is made available under aCC-BY 4.0 International license.

signals showing relatively diffuse expression, (viii) signals showing locally concentrated expression, and (ix) noise.

E. The spatial distribution of feature 266 in the Perilipin protein channel is illustrated for samples under fasted, high fat diet (HFD), and *ad lib* conditions. Cells displaying high Perilipin 266 values contain high amounts of concentrated perilipin clusters. Notably, pre-normalized values for the Perilipin 266 feature are considerably higher in the HFD sample.

bioRxiv preprint doi: https://doi.org/10.1101/2024.11.18.624217; this version posted February 17, 2025. The copyright holder for this preprint (which was not certified by peer review) is the author/funder, who has granted bioRxiv a license to display the preprint in perpetuity. It is made available under aCC-BY 4.0 International license.

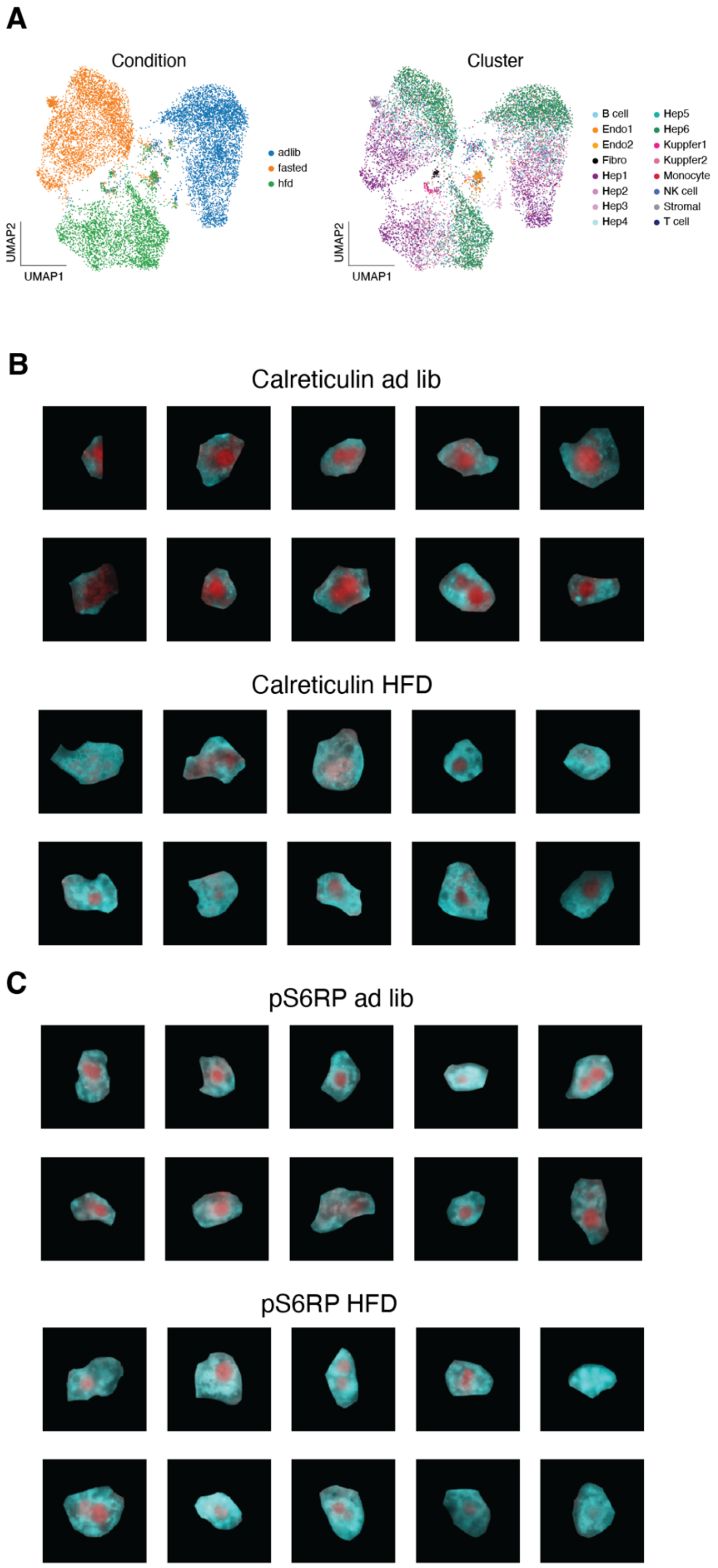

bioRxiv preprint doi: https://doi.org/10.1101/2024.11.18.624217; this version posted February 17, 2025. The copyright holder for this preprint (which was not certified by peer review) is the author/funder, who has granted bioRxiv a license to display the preprint in perpetuity. It is made available under aCC-BY 4.0 International license.

Figure S7: Changes in gene expression and morphology with physiological state

A. UMAP of individual cells measured by 10X Flex from mice either with *ad lib* diet, overnight fasting, or 1 month high fat diet (HFD), colored by condition (left) or cell-type and subtype identity (right).
B. Examples of calreticulin morphology in cells under *ad lib* or HFD conditions.
C. Examples of pS6RP morphology in cells under *ad lib* or HFD conditions.

bioRxiv preprint doi: https://doi.org/10.1101/2024.11.18.624217; this version posted February 17, 2025. The copyright holder for this preprint (which was not certified by peer review) is the author/funder, who has granted bioRxiv a license to display the preprint in perpetuity. It is made available under aCC-BY 4.0 International license.

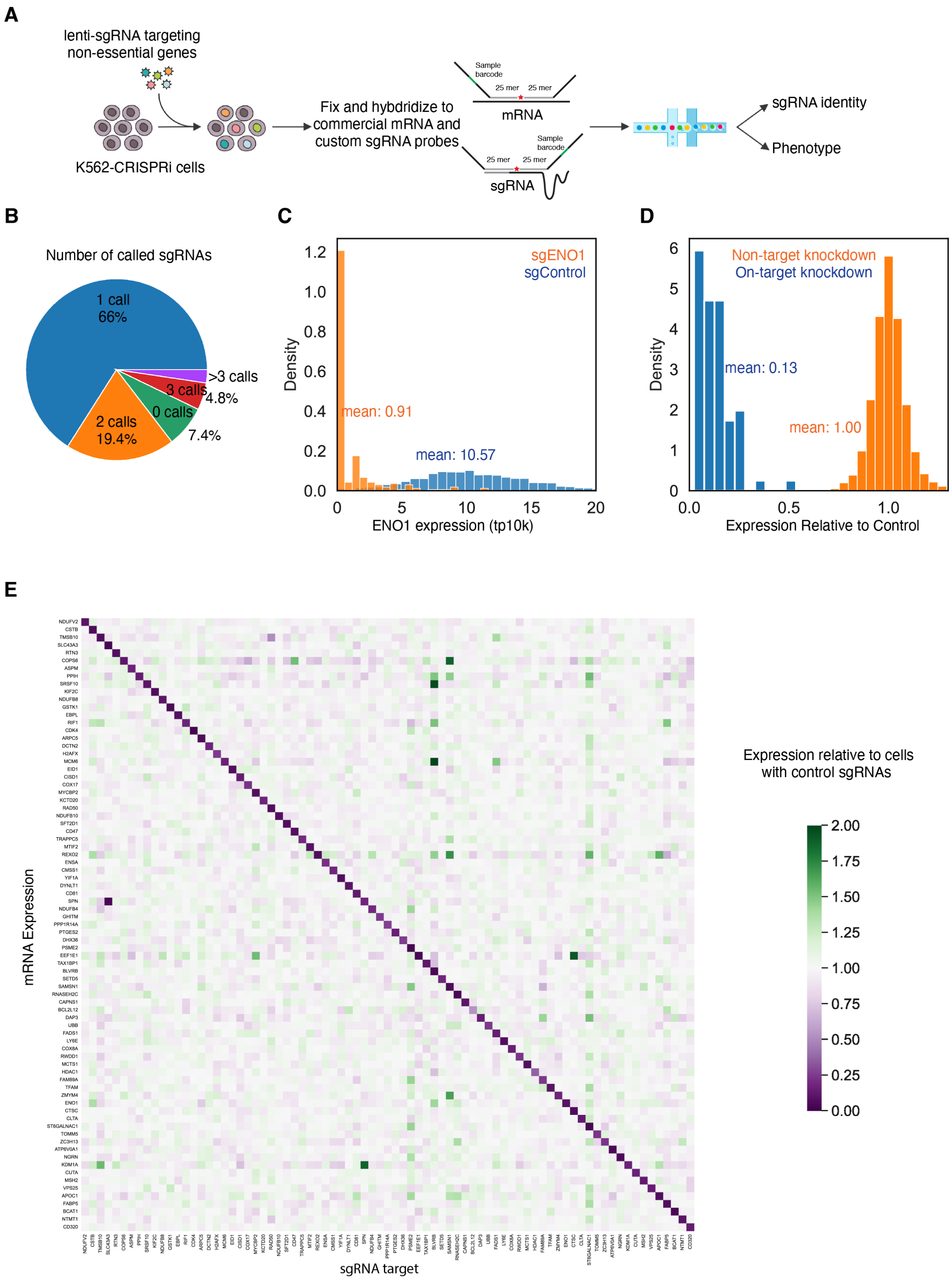

bioRxiv preprint doi: https://doi.org/10.1101/2024.11.18.624217; this version posted February 17, 2025. The copyright holder for this preprint (which was not certified by peer review) is the author/funder, who has granted bioRxiv a license to display the preprint in perpetuity. It is made available under aCC-BY 4.0 International license.

Figure S8: Validation of fixed cell Perturb-seq with CRISPRi

A. Diagram of K562-CRISPRi Perturb-seq validation experiment
B. Pie chart of the number of called sgRNA per cell.
C. Histogram representing *ENO1* expression in cells with an sgRNA targeting *ENO1* or in cells with control sgRNAs.
D. Histogram representing on-target knockdown and off-target knockdown, averaged across all targets in the experiment. On-target knockdown is defined as the expression of the target gene in cells with each corresponding sgRNA, relative to cells with control sgRNAs. Off-target knockdown is defined as the expression of each of other genes targeted in the experiment (not targeted in that cell), relative to expression of those genes in cells with control sgRNAs.
E. Heat map representation of average expression of each of the indicated genes in cells with each of the indicated sgRNAs, relative to expression of those genes in cells with control sgRNAs.

bioRxiv preprint doi: https://doi.org/10.1101/2024.11.18.624217; this version posted February 17, 2025. The copyright holder for this preprint (which was not certified by peer review) is the author/funder, who has granted bioRxiv a license to display the preprint in perpetuity. It is made available under aCC-BY 4.0 International license.

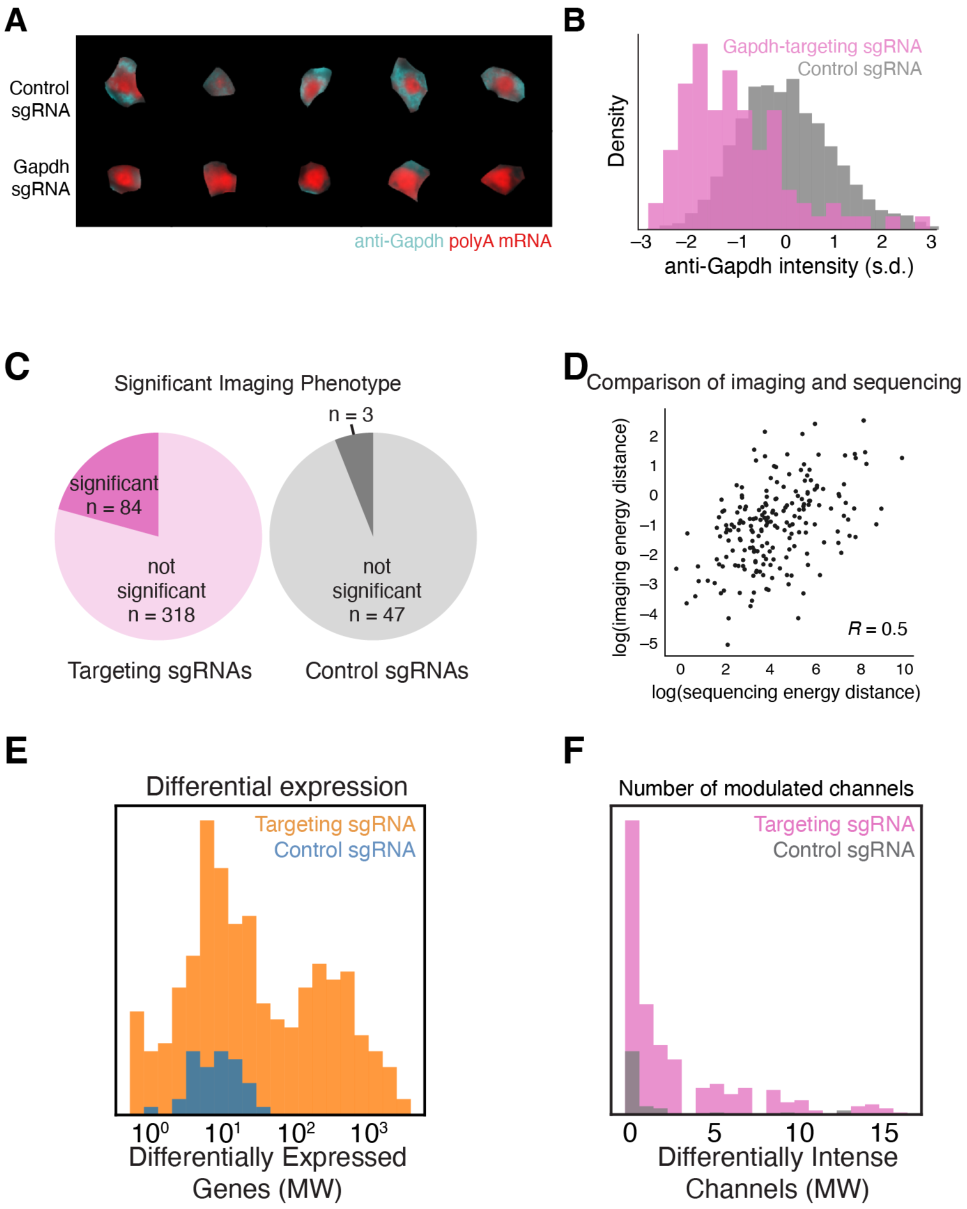

Figure S9: Additional analyses of perturbation data

A. Unbiased sampling of cells with control sgRNAs and sgRNAs targeting *Gapdh*. The fluorescence micrographs show anti-GAPDH and polyA FISH channels.
B. Histogram comparing anti-Gapdh intensity in called cells with a control sgRNA and called cells with *Gapdh*-targeting sgRNAs, from the imaging dataset.
C. Pie charts showing the number of targeting (left) and control (right) sgRNAs that caused a significant transcriptional phenotype, as measured by a Holm-Sidak-corrected energy distance permutation test ($p < 0.05$), in the imaging dataset.

bioRxiv preprint doi: https://doi.org/10.1101/2024.11.18.624217; this version posted February 17, 2025. The copyright holder for this preprint (which was not certified by peer review) is the author/funder, who has granted bioRxiv a license to display the preprint in perpetuity. It is made available under aCC-BY 4.0 International license.

D. Scatterplot comparing the energy distance vs control cells for each knockout in the imaging and Perturb-seq datasets.
E. Histogram representing the number of differentially expressed genes for each perturbation, from the sequencing experiment. Differential gene expression reflects Benjamini-Hochberg-corrected, Mann-Whitney $p < 0.05$, versus cells with control sgRNAs.
F. Histogram representing the number of imaging channels (proteins or RNAs) exhibiting differentially intense signals foreach perturbation, from the imaging experiment. Differentially intense signal reflects Benjamini-Hochberg-corrected, Mann-Whitney $p < 0.05$, versus cells with control sgRNAs.

Supplementary Tables:

- **Table S1: Fluorescent readout oligonucleotide bits used for RCA-MERFISH**
  - The sequences and colors of the fluorescent readout oligonucleotide bits used for all RCA-MERFISH imaging.
- **Table S2: RCA-MERFISH mRNA Library**
  - The targets and sequences of the padlock probes used for the mRNA measurements in RCA-MERFISH.
- **Table S3: RCA-MERFISH Oligo-Antibody + Abundant RNA Panel**
  - The antibodies used for multiplexed oligo-antibody immunofluorescence, as well as the abundant RNAs targeting with sequential FISH.
- **Table S4: RCA-MERFISH Abundant RNA Probes**
  - Sequences of the oligonucleotide probes used for FISH targeting abundant RNAs.
- **Table S5: RCA-MERFISH Imaging Experiment Rounds, WT Animals**
  - Description of the imaging rounds in the RCA-MERFISH experiments conducted in WT animals.
- **Table S6: Liver Perturbation Library**
  - Targets, sgRNA sequences, and barcode sequences used to generate the genetic mosaic livers that we analyzed with imaging and Perturb-seq.
- **Table S7: RCA-MERFISH Perturbation Barcode Library**
  - The targets and sequences of the padlock probes used for the perturbation barcode measurements in RCA-MERFISH.
- **Table S8: RCA-MERFISH Imaging Experiment Rounds, Mosaic Animals**
  - Description of the imaging rounds in the RCA-MERFISH experiments conducted in genetic mosaic animals.
- **Table S9: Fixed Cell Perturb-seq sgRNA Probes**
  - Sequences of sgRNA-targeting probes used in fixed-cell Perturb-seq.
